# MiR-135-5p promotes osteoblast differentiation by targeting HIF1AN in MC3T3-E1 cells

**DOI:** 10.1186/s11658-019-0177-6

**Published:** 2019-08-08

**Authors:** Nuo Yin, Longzhang Zhu, Liang Ding, Junjie Yuan, Li Du, Mingmang Pan, Feng Xue, Haijun Xiao

**Affiliations:** Department of Orthopaedics, Shanghai Fengxian District Central Hospital, No. 6600, Nanfeng Highway, Shanghai, 201499 China

**Keywords:** miR-135, Osteoblast differentiation, Calcification, Hypoxia-inducible factor 1 α inhibitor

## Abstract

**Background:**

MicroRNAs (miRNAs or miRs) serve crucial roles in the progression of osteoporosis. This study investigated the role and specific molecular mechanism of miR-135-5p in regulating osteoblast differentiation and calcification.

**Methods:**

Bone morphogenetic protein 2 (BMP2) was employed to interfere with the differentiation of MC3T3-E1. Then, miR-135-5p mimic or miR-135-5p inhibitor was transfected into MC3T3-E1, and quantitative RT-PCR was used to measure the expression of miR-135-5p. The expressions of runt-related transcription factor 2 (Runx2), osterix (OSX), osteopontin (OPN), and osteocalcin (OCN) were determined using western blot. Alkaline phosphatase (ALP) activity was measured using an appropriate kit assay. Calcium nodule staining was evaluated with alizarin red staining. A luciferase reporter assay was used to verify the target of miR-135-5p. Hypoxia-inducible factor 1 α inhibitor (HIF1AN) overexpression was applied to investigate its own role in the mechanism and a miR-135-5p rescue experiment was also performed.

**Results:**

Overexpression of miR-135-5p promoted osteogenic differentiation and calcification, as shown by the increase in ALP activity, calcification and osteogenic marker levels, including Runx2, OSX, OPN and OCN. Knockdown of miR-135-5p yielded the opposite results. HIF1AN was confirmed as a direct target of miR-135-5p. HIF1AN overexpression inhibited osteogenic differentiation and calcification while miR-135-5p reversed these effects.

**Conclusions:**

These results indicate that miR-135-5p might have a therapeutic application related to its promotion of bone formation through the targeting of HIF1AN.

## Background

Osteoporosis is a chronic systemic bone disease manifesting as lower bone mass and a disorder of the bone structure, which eventually contributes to the risk of fracture [1, 2]. Osteogenic differentiation is a key factor in bone regeneration. Clarifying the regulatory mechanisms for osteoblast differentiation and calcification is very important for the improvement of the treatments for bone-related diseases [3]. A better understanding of the molecular mechanisms that govern osteogenesis might provide us with new perspectives on the treatment of osteoporosis. Therefore, it is of clinical importance that novel therapeutic targets and biomarkers associated with osteoporosis are identified.

MicroRNAs (miRNAs) are small noncoding RNAs with between 20 and 24 nucleotides. They have been shown to regulate gene expression at the post-transcriptional level in numerous biological processes [4, 5]. Mounting evidence indicates that they can regulate bone formation at all stages and are involved in osteoporosis and other kinds of bone disease [6–8]. A previous study showed that miR-449c-5p inhibits the osteogenic differentiation of human valve interstitial cells (VICs) through the Smad4-mediated pathway [9]. miR-210 ameliorates post-menopausal estrogen deficiency-related osteoporosis by increasing VEGF expression and osteoblast differentiation [10]. These findings show that miRNAs obviously impact osteoblast differentiation. Furthermore, it has been well documented that miR-135 and miR-203 impair the progression of breast cancer and metastatic bone disease through their targeting of Runx2 [11]. Importantly, miR-135 was considered to be an osteogenesis-related miRNA that was upregulated during the osteogenesis of rat adipose-derived stem cells [12]. However, the effect of miR-135 on preosteoblasts remains to be elucidated.

Emerging evidence supports the idea that hypoxia-inducible factor 1 α (HIF-1α) can promote osteoblast differentiation [13, 14]. HIF-1α inhibitor (HIF1AN) is a well-known negative modulator of HIF-1α. A previous study suggested that miR-135b affects the HIF1AN protein level. This is attributed to its binding to HIF1AN 3′-UTR [15]. However, there are no reports of a role for HIF1AN in osteoblast differentiation.

Here, we investigate the relationship between miR-135 and HIF1AN in BMP2-induced MC3T3-E1. Our results may be useful in enhancing new bone formation and designing treatments for pathological bone loss.

## Materials and methods

### Cell culture and differentiation of MC3T3-E1 cells

MC3T3-E1 cells were obtained from the Cell Bank of the Chinese Academy of Sciences and cultured in α-minimum essential medium (MEM) supplemented with 10% fetal bovine serum (FBS; Thermo Fisher Scientific) in a humidified 10% CO_2_ atmosphere at 37 °C. The cells were then seeded into 6-well plates at a density of 1 × 10^6^ cells/well. When they reached 80% confluence, they were transferred to differentiation medium (DM) containing 10% FBS with 50 μg/ml ascorbic acid and 4 mM β-glycerol phosphate for culture. Fresh medium was applied every three days.

MC3T3-E1 is a murine pre-osteoblast cell line that can differentiate into osteoblasts when stimulated with BMP-2, which is a well-accepted model for investigating the osteogenic differentiation [16]. For the experiments, BMP2 (300 ng/ml) was added to the DM and replaced every 72 h in the BMP2 intervention group. For studies of osteoblast differentiation, the cells were grown for 0 to 14 days in DM with or without BMP-2.

### Cell transfection

We obtained the sequence of the mature miR-135-5p from miRBase (http://www.mirbase.org/). The miR-135 mimic or miR-135 inhibitor and their negative control (NC) were chemically synthesized by Ribobio. When the cells grew to 70% confluence, they were transfected with miR-135 mimic or miR-135 inhibitor and incubated at 37 °C.

### Cell counting Kit-8 (CCK-8) assay

Cell viability was measured using the Cell Counting Kit-8 assay (CT-K; Shanghai Yi Sheng Biotechnology) according to the manufacturer’s instruction. At 24 h post-transfection, 100 μl cell suspensions were seeded into 96-well plates at a density of 8 × 10^3^ cells/well and incubated at 37 °C for 6 h. At the indicated time points (0 d, 1 d, 2 d, 3 d, 5 d, 7 d and 14 d), 10 μl CCK-8 solution was added to each well. Following incubation at 37 °C for 1 h, othe ptical density (OD) value was measured at 450 nm on a microplate reader. Each experiment was performed in triplicate.

### Alkaline phosphatase (ALP) activity assay

ALP activity was assessed on day 14 after osteogenic induction. ALP activity was examined using an ALP activity kit according to the manufacturer’s protocol (Beyotime). The absorbance was examined at 405 nm.

### Calcium nodule staining (alizarin red staining)

The third generation of cells was cultured for two weeks and then mineralized to form opaque calcified nodules. The cell samples were washed 1 or 2 times with PBS, fixed with 95% ethanol for 10 min, washed 1 or 2 times with PBS again, covered, and stained with 0.1% alizarin red solution for 10 min. Finally, they were rinsed with PBS and observed under an inverted light microscope.

### Luciferase reporter assay

The target genes of miR-135-5p were predicted using TargetScan database version 7.1 (http://www.targetscan.org/vert_71/). Wild-type (WT) and mutant-type (MUT) HIF1AN 3′-UTR luciferase reporter vectors were designed. MiR-135-5p mimics or the mimic control were co-transfected with constructed the WT or MUT luciferase reporter vector into MC3T3-E1 cells using Lipofectamine 2000 (Thermo Fisher Scientific). Luciferase activity was assessed using a Dual Luciferase Reporter Gene Assay Kit (RG027; Beyotime) after cell transfection for 48 h.

### Quantitative RT-PCR

Total RNA was isolated using TRIzol reagent (Invitrogen) according to the manufacturer’s instructions. Then cDNA was synthesized using a RevertAid First Strand cDNA Synthesis Kit (K1622, Thermo Fermentas). Quantitative PCR was performed using iTaq Universal SYBR Green Supermix (Bio-Rad). The sequence for miR-135-5p was 5′-UAUGGCUUUCUUUUCCUGUGUG-3′. The primers used were: miR-135-5p, forward 5′-AGCATAATACAGCAGGCACAGAC-3′, reverse 5′-AAAGGTTGTTCTCCACTCTCTCAC-3′; HIF1AN, forward 5′-GTACTGGTGGCATCACATAGAG-3′, reverse 5′-CTGATGGGCTTTGAGAGGATATT-3′; GAPDH, forward 5′-AGCTTCGGCACATATTTCATCTG-3′, reverse 5′-CGTTCACTCCCATGACAAACA-3′; and U6, forward 5′-TTGACTCCACAAAAGGGAAGAAG-3′, reverse 5′-TCCAGAGGTCTGTTGAATCCG-3′. GAPDH or U6 was used as an internal control. CTRP3 expression was analyzed using the 2^-△△Ct^ method.

### Western blotting assay

The MC3T3-E1 cells were seeded at 2 × 10^6^ cells/well in 6-well plates and cultured for 24 h before the experiment. Cells were harvested and lysed on ice in RIPA Lysis Buffer (Beyotime). The bicinchoninic acid assay (Kaiji) was employed to measure the concentration of protein. 50 μg proteins were isolated using SDS-PAGE. Subsequently, proteins were transferred to polyvinylidene difluoride membranes (PVDF; Millipore). The membranes were blocked with 5% non-fat milk and incubated overnight with primary antibodies at 4 °C. The membranes were washed three times (0.1% Tween 20 in PBS, 10 min at one time) and incubated with HRP-labeled goat anti-mouse IgG (H + L) antibody (A0216; Beyotime) at room temperature for 2 h. The blots were developed with an enhanced chemiluminescence reagent and analyzed using ImageJ software. Anti-Runx2 (8486S) antibody was obtained from Cell Signaling Technology. Anti-HIF1AN (D123653), anti-OSX (D161992), anti-OPN (D221078) and anti-GAPDH (D110016) were from Sangon Biotech. Anti-OCN (sc-73464) was from Santa Cruz Biotechnology.

### Statistical analysis

All results were confirmed in at least three independent experiments and all statistical analyses were conducted using SPSS 20.0 software. The results were expressed as the means ± standard deviation. Quantitative data were compared using one-way analysis of variance and Student’s t-test. A significance level of *p* < 0.05 was adopted for all analyses.

## Results

### miR-135-5p is upregulated in MC3T3-E1 osteoblasts following treatment with BMP2

**Fig. 1:**
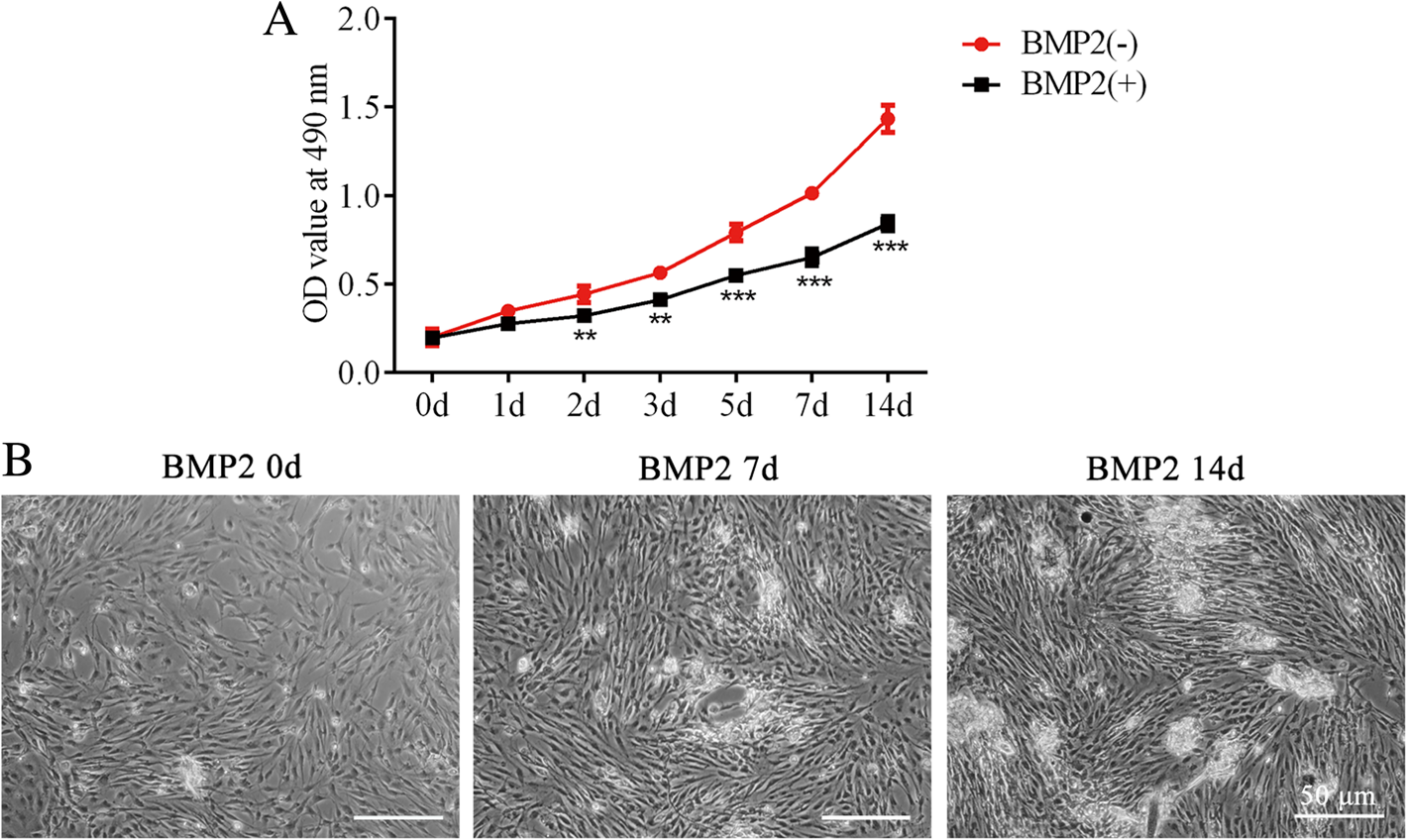
BMP2 induces osteoblast differentiation of MC3T3-E1 cells. **a** – Cell viability was detected using the CCK-8 assay after MC3T3-E1 cells were treated with 300 ng/ml BMP2. **p < 0.01, ***p < 0.001 vs. BMP (−). **b** – Cell morphology of MC3T3-E1 0, 7 and 14 days after MC3T3-E1 cells were treated with 300 ng/ml BMP2

After being cultured in BMP2 for 14 days, the MC3T3-E1 cells displayed a lower proliferative capacity than those cultured in DM without BMP2 (Fig. 1a). This result was in accordance with results of a previous study [17].

**Fig. 2:**
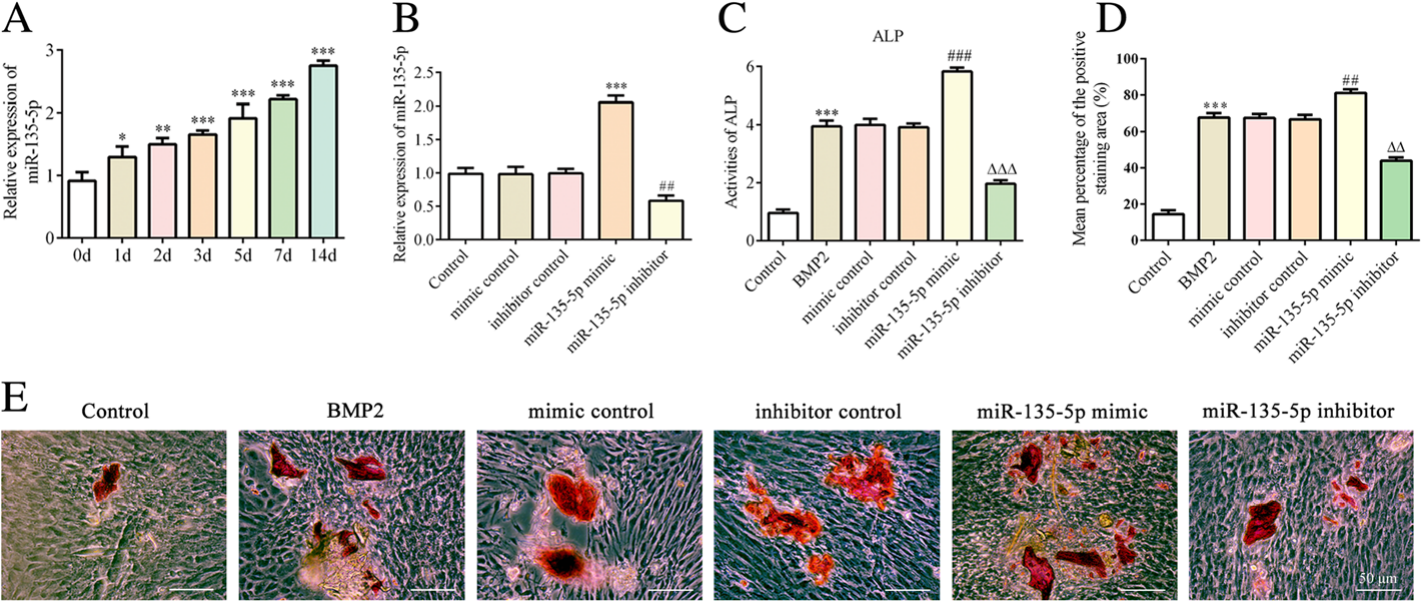
The levels of ALP and calcification after miR-135-5p overexpression or knockdown during osteoblast differentiation of MC3T3-E1 cells. **a** – The expressions of miR-135-5p after MC3T3-E1 cells were treated with 300 ng/ml BMP2 for osteogenic induction (determined using quantitative RT-PCR). *p < 0.05, **p < 0.01, ***p < 0.001 vs. day 0. **b** – The expression of miR-135-5p after MC3T3-E1 cells were transfected with miR-135-5p mimic or miR-135-5p inhibitor (determined using quantitative RT-PCR). ***p < 0.001 vs. mimic control; ^##^*p* < 0.01 vs. inhibitor control. **c** – The level of ALP was measured using an ALP assay kit. **d** – The area stained with alizarin red staining was quantified. **e** – The level of calcification was measured using alizarin red staining. ***p < 0.001 vs. control; ^##^*p* < 0.01, ^###^*p* < 0.001 vs. mimic control; ^△△^*p* < 0.01, ^△△△^*p* < 0.001 vs. inhibitor control

Concurrently, we recorded the cell growth situation of MC3T3-E1 on day 0, 7 and 14 in the presence of BMP2 (Fig. 1b). Then, to determine whether miR-135-5p is involved in the regulation of osteoblast differentiation, the expression of miR-135-5p in the presence of BMP2 was measured via quantitative RT-PCR. An obvious increasing trend was found at each time point over the 14 days, and it was the highest on day 14 (Fig. 2a). These dates indicate that miR-135-5p is upregulated during osteoblast differentiation of MC3T3-E1 cells.

### miR-135-5p promotes the osteoblast differentiation of MC-3 T3-E1 cells

To investigate the exact effect of miR-135-5p on osteoblast differentiation, MC-3 T3-E1 cells were treated with an miR-135-5p mimic or miR-135-5p inhibitor. These treatments respectively upregulated or downregulated miR-135-5p in MC3T3-E1 cells (Fig. 2b).

We then evaluated the levels of ALP activity and calcification, which are phenotypic markers of osteogenic differentiation. As shown in Fig. 2c–e, MC3T3-E1 cells undergoing osteoblast differentiation exhibited significantly higher ALP activity and calcification than the controls. Following treatment with the miR-135-5p mimic, the levels of ALP activity and calcification were markedly higher than for the mimic control group. By contrast, cells treated with the miR-135-5p inhibitor showed the opposite results.

**Fig. 3:**
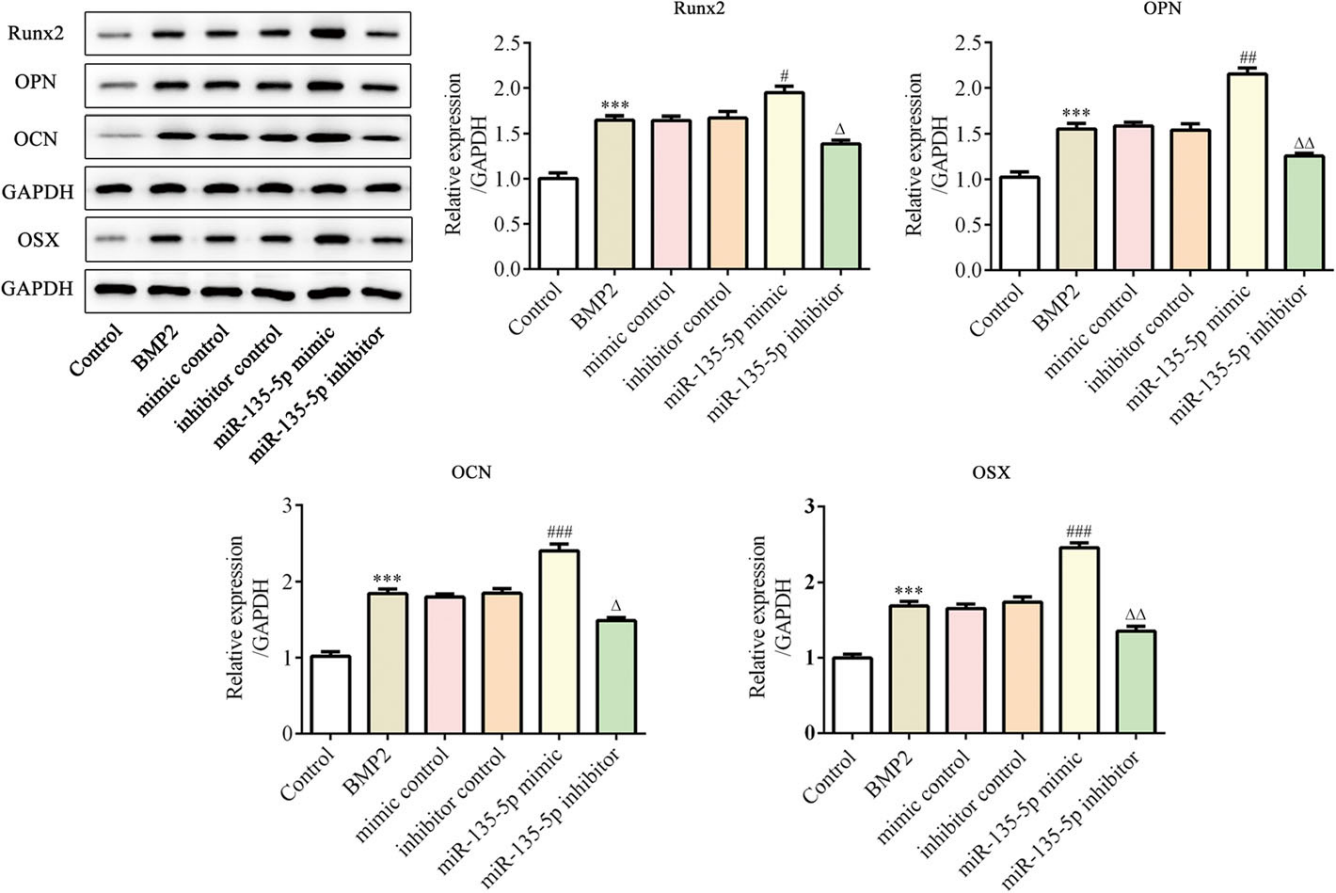
The expression levels of osteogenesis-related marker proteins after miR-135-5p overexpression or knockdown during osteoblast differentiation of MC3T3-E1 cells. The expressions of Runx2, OPN, OCN and OSX were determined using western blot. ***p < 0.001 vs. control; ^#^*p* < 0.05, ^##^*p* < 0.01, ^###^*p* < 0.001 vs. mimic control; ^△^*p* < 0.05, ^△△^*p* < 0.01 vs. inhibitor control

At the same time, the expression levels of osteoblast differentiation-associated proteins were measured using western blot. We found that the levels of Runx5, OSX, OPN and OCN increased following incubation with BMP2. The miR-135-5p mimic promoted the expressions of those proteins, whereas the miR-135-5p inhibitor suppressed them (Fig. 3). These results indicate that miR-135-5p promotes osteoblast differentiation of MC-3 T3-E1 cells.

### HIF1AN is a target gene of miR-135-5p

**Fig. 4:**
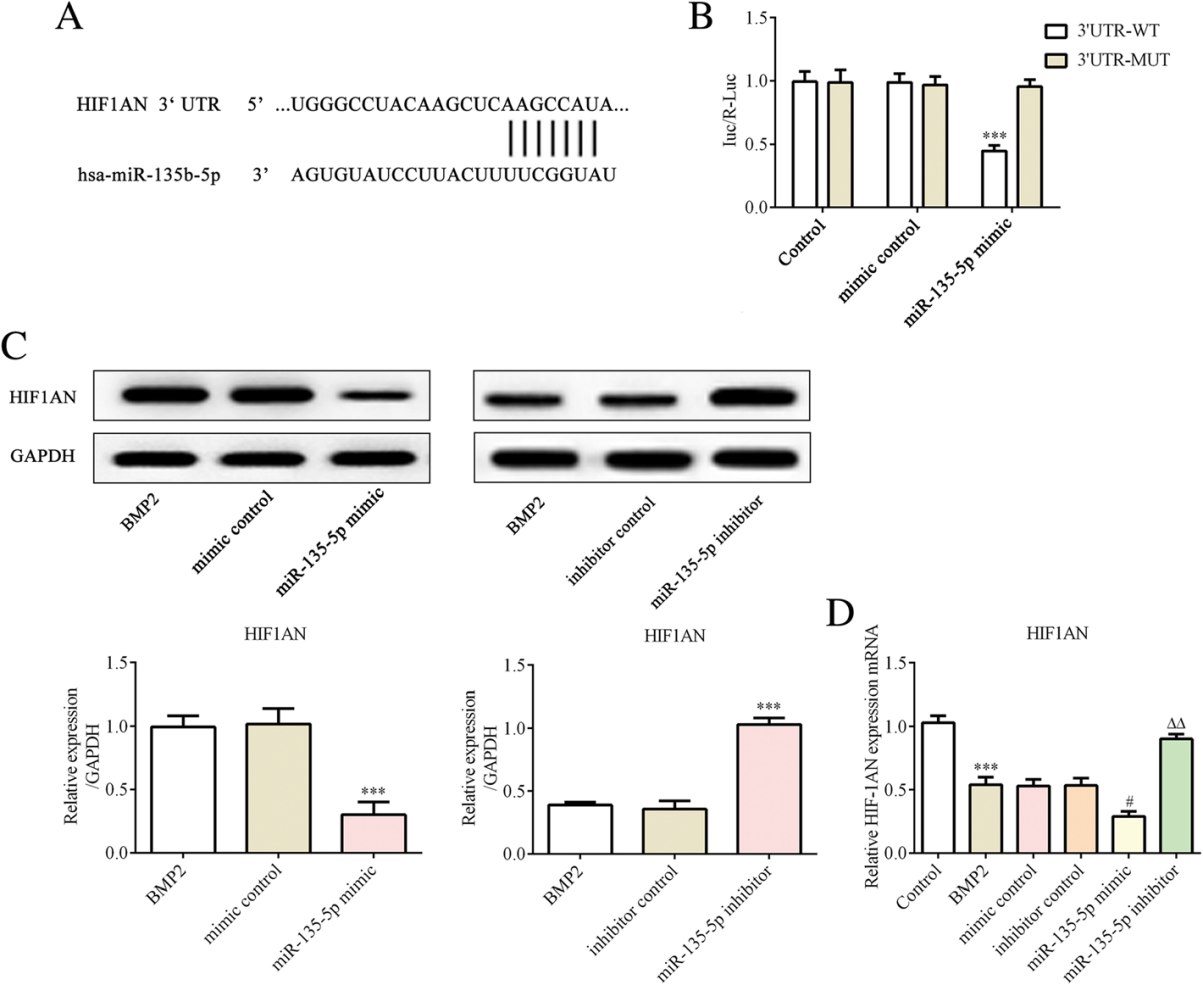
HIF1AN is a target gene of miR-135-5p. **a** – The predicted binding site between the miR-135-5p and HIF1AN, determined using bioinformatics analysis. **b** – The luciferase activity of HIF1AN-WT and HIF1AN-MUT treated with miR-135-5p mimic or mimic control. ***p < 0.001 vs. 3′-UTR-MUT. **c** – The expression of HIF1AN was determined using western blot. ***p < 0.001 vs. mimic control. **d** – The expression of HIF1AN was determined via RT-qPCR. ***p < 0.001 vs. control; ^#^*p* < 0.05 vs. mimic control; ^△△^*p* < 0.01 vs. inhibitor control

To elucidate the underlying molecular mechanisms of miR-135-5p in osteoblast differentiation of MC3T3-E1 cells, we searched for the potential target sites of miR-135-5p in the Target Scan database. HIF1AN was predicted to be a potential target of miR-135-5p (Fig. 4a).

A luciferase activity assay was employed to validate the targeting of HIF1AN by miR-135-5p. We constructed and verified a wild-type HIF1AN 3′-UTR luciferase reporter plasmid and the mutant, which were then used for co-transfection with miR-135-5p mimic or the mimic control into MC3T3-E1 cells. The cells co-transfected with the wild-type 3′-UTR and the miR-135-5p mimic presented significantly decreased luciferase activity (Fig. 4b). In addition, we used western blot and quantitative RT-PCR to further evaluate the target. We found that the protein and mRNA expressions of HIF1AN were lower in miR-135-5p mimic group, whereas the expression of HIF1AN was promoted in the miR-135-5p inhibitor group (Fig. 4c and d). These results indicate that HIF1AN is negatively regulated by miR-135-5p.

### Overexpression of HIF1AN alleviates the stimulatory effect of miR-135-5p on osteogenesis

**Fig. 5:**
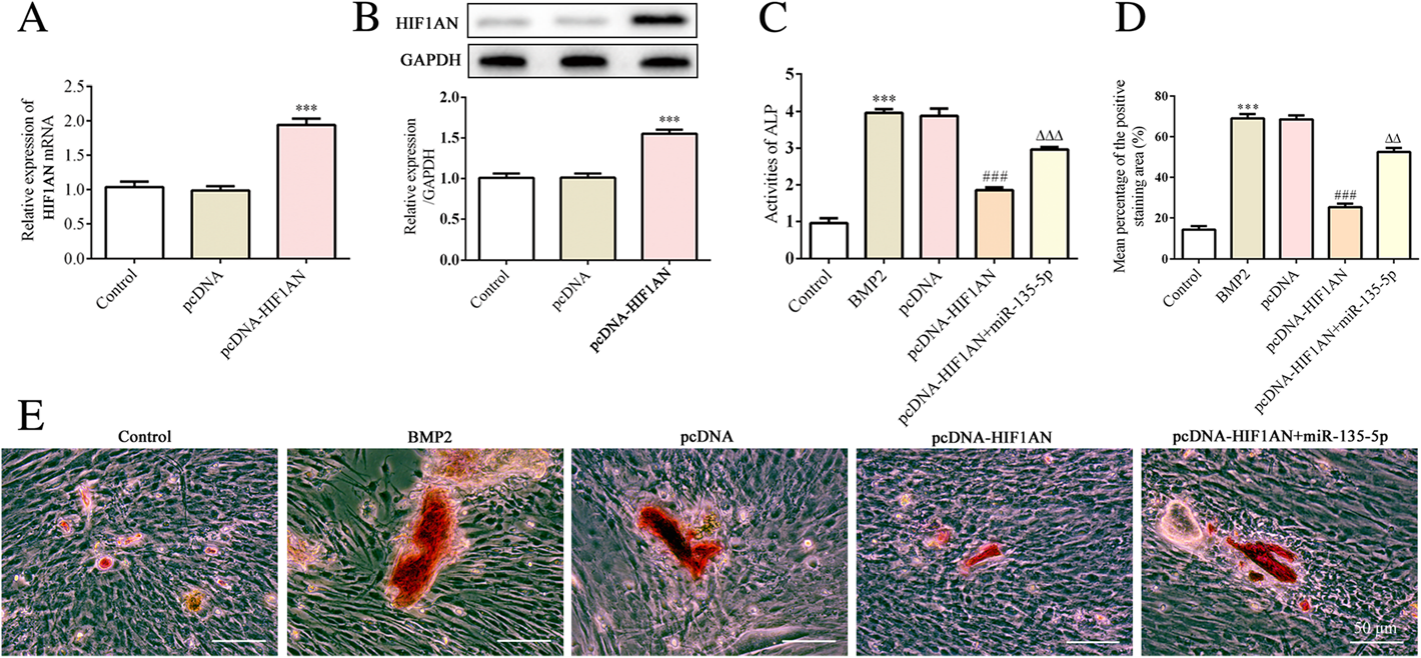
The levels of ALP and calcification after HIF1AN overexpression or knockdown during osteoblast differentiation of MC3T3-E1 cells after incubation in DM supplemented with BMP2 for 14 days. **a** and **b** – The expression of HIF1AN following MC3T3-E1 cells being transfected with HIF1AN pcDNA3.1 or pcDNA3.1 was measured via quantitative RT-PCR (**a**) and western blot (**b**). ***p < 0.001 vs. pcDNA. **c** – The level of ALP was measured using an ALP assay kit. **d** – The area stained with alizarin red staining was quantified. **e** – The level of calcification was measured using alizarin red staining. ***p < 0.001 vs. control; ^###^*p* < 0.001 vs. pcDNA; ^△△^*p* < 0.01, ^△△△^*p* < 0.001 vs. pcDNA-HIF1AN + miR-135-5p

**Fig. 6:**
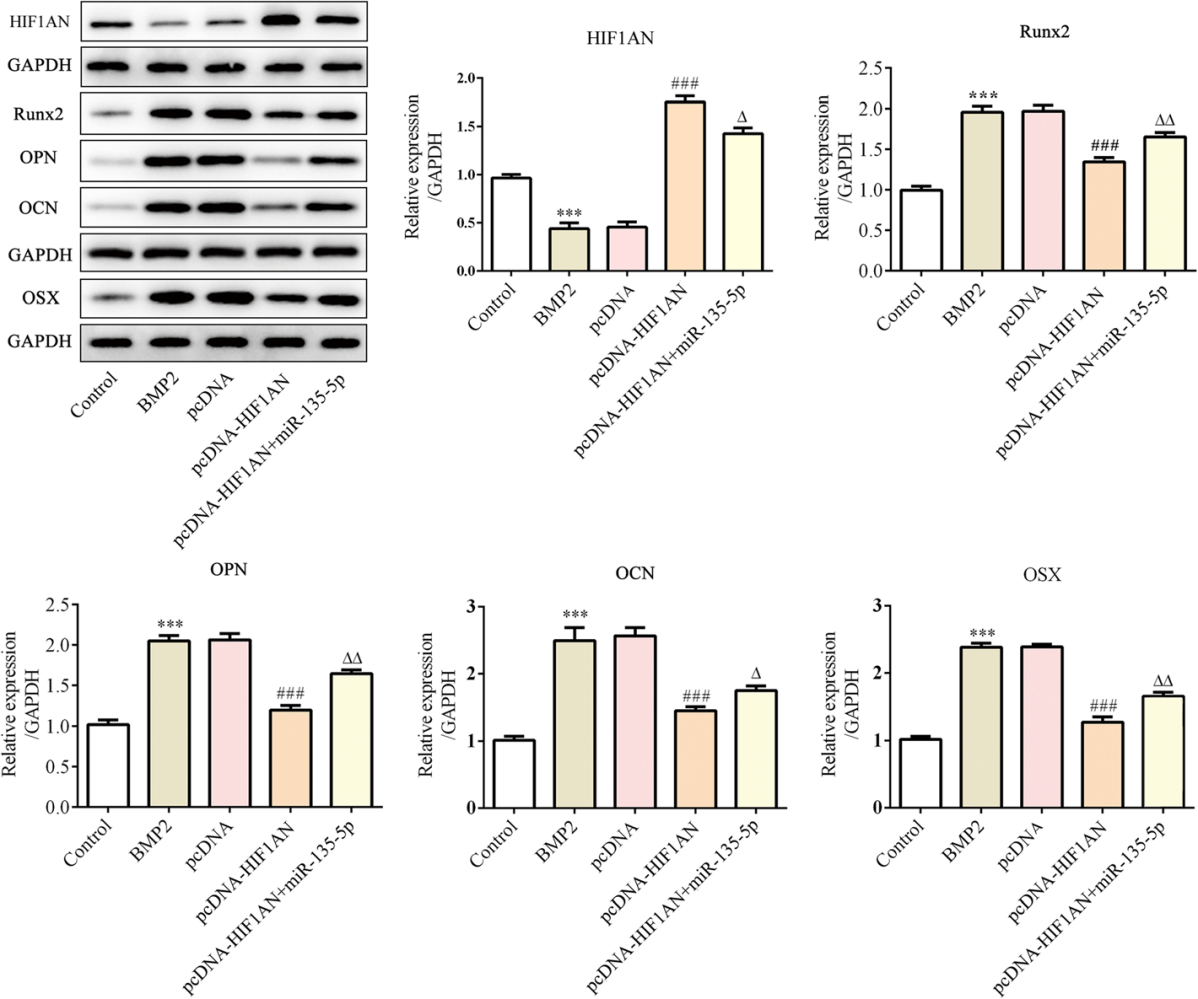
MiR-135-5p promotes osteoblast differentiation of MC3T3-E1 cells by targeting HIF1AN after incubation in DM supplemented with BMP2 for 14 days. The protein levels of Runx2, OPN, OCN and OSX in MC3TC-E1 cells subjected to the indicated treatments were determined using western blotting. ***p < 0.001 vs. control; ^###^*p* < 0.001 vs. pcDNA; ^△^*p* < 0.05, ^△△^*p* < 0.01 vs. pcDNA-HIF1AN + miR-135-5p

To further explore whether HIF1AN alleviated the effect of miR-135-5p on osteoblast differentiation, HIF1AN pcDNA3.1 or pcDNA3.1 was transfected into MC3T3-E1 cells and subsequently treated with BMP2 for 14 days. The expressions of HIF1AN mRNA and protein were respectively determined using quantitative RT-PCR and western blot. As shown in Fig. 5a and b, overexpression of HIF1AN was successfully achieved. Cells treated with both HIF1AN pcDNA3.1 and miR-135-5p mimic presented higher ALP activity and calcification than cells transfected with HIF1AN pcDNA3.1 alone (Fig. 5c–e). Moreover, the expressions of Runx5, OSX, OPN and OCN were upregulated following treatment with HIF1AN pcDNA3.1 and miR-135-5p mimic compared to the levels in the cells treated with HIF1AN pcDNA3.1 alone (Fig. 6). These results indicate that overexpression of HIF1AN alleviates the stimulatory effect of miR-135-5p on osteogenesis.

## Discussion

Our study demonstrated that miR-135-5p promotes osteoblast differentiation and mineralization of MC3T3-E1 cells through binding to the 3′-UTR sites of HIF1AN mRNA, which hampers its translation. This is the first demonstration of the role and underlying mechanisms of miR-135-5p during osteogenesis.

Numerous studies have shown that miRNAs could act as key modulators in osteoblastic differentiation. MiR-141 and miR-200a are involved in osteogenic differentiation through their targeting of Dlx5 in MC3T3-E1 cells [18]. miR-378 can promote osteoblast differentiation by targeting BMP2 [19]. In addition, miR-764-5p promotes osteoblast differentiation through inhibition of CHIP/STUB1 expression [20]. It has been well documented that miR-135 is an osteogenesis-related microRNA, and that the expression level of miR-135 increases during the osteogenesis of rat adipose-derived stem cells [12].

Here, we found that miR-135-5p was upregulated following induction of BMP2 in MC3T3-E1 cells. miR-135-5p overexpression significantly enhanced ALP activity and extracellular matrix calcium deposition, whereas knockdown of miR-135 suppressed these processes. These findings were in accordance with those from a previous study about the function of miR-135 in osteogenic differentiation [21].

Mounting evidence supports the idea that Runx2 serves as a critical osteoblast lineage-determining transcription factor that is involved in directing osteoblastic differentiation [22]. Runx2 appears to be the master gene in osteogenesis as it is able to induce the expressions of OPN, OCN and OSX, which are all osteogenesis-related markers and required for terminal osteoblast differentiation. In our study, miR-135-5p overexpression upregulated the expressions of Runx2, OPN, OCN and OSX, whereas miR-135-5p knockdown downregulated the expression of the above proteins, which was consistent with the results of previous studies [12]. These results indicate that miR-135-5p could promote osteogenic differentiation.

It was reported that activation of the HIF-1α signaling pathway upregulated osteogenic differentiation-related genes in mesenchymal stem cells [23]. Emerging evidence indicates that increased HIF-1α expression can promote the osteoblast differentiation of marrow-derived stem cells [24]. HIF1AN was considered to be an important inhibitor that can interact with HIF-1α. Considerable evidence has shown that HIF1AN plays critical roles in the differentiation of various tissues. For example, miR-455 could regulate brown adipocyte differentiation by targeting HIF1AN [25]. In addition, in the epidermis and corneal epithelium, miR-31 targets HIF1AN, leading to a more differentiated phenotype, and HIF1AN hydroxylates Notch [26, 27]. Importantly, a previous study suggested that miR-135b affects the protein level of HIF1AN, which is attributed to its binding to HIF1AN 3′-UTR [15]. However, there has been no report focusing on miR-135-5p regulating osteogenic differentiation through the targeting of HIF1AN.

In this study, we discovered that HIF1AN was the direct target of miR-135-5p and that overexpression of HIF1AN reduced the levels of ALP activity, calcium deposition, and OPN, OCN and OSX, whereas the miR-135-5p mimic reversed these results. Our results indicate that MiR-135-5p promotes osteoblast differentiation by targeting HIF1AN.

## Conclusions

We here provide evidence that miR-135-5p can induce osteogenesis by sponging HIF1AN. Therefore, this study also provides new insights into the roles and regulatory mechanisms of miRNAs in osteogenic differentiation. Our results suggest that therapeutic approaches targeting miR-135-5p could be useful in enhancing new bone formation and the treatment of pathological bone loss.

## Abbreviations

ALP: Alkaline phosphatase
BMP2: Bone morphogenetic protein 2
HIF1AN: Hypoxia-inducible factor 1 α inhibitor
HIF-1α: Hypoxia-inducible factor 1 α
miRNA/miRs: microRNAs
MUT: Nutant-type
OCN: Osteocalcin
OPN: Osteopontin
OSX: Osterix
Runx2: Runt-related transcription factor 2
WT: Wild-type
